# IMAGE001: A new livestock multispecies SNP array to characterize genomic variation in European livestock gene bank collections

**DOI:** 10.1111/age.70039

**Published:** 2025-09-18

**Authors:** R. P. M. A. Crooijmans, R. Gonzalez Prendes, L. Colli, M. Del Corvo, M. Barbato, E. Somenzi, G. Tosser‐Klopp, G. Meszaros, P. Ajmone‐Marsan, S. Weigend, B. Wallner, M. E. McCue, L. Orlando, D. Bradley, S. J. Hiemstra, D. Schokker, N. Peynot, A. Stella, G. Restoux, M. A. M. Groenen, M. Tixier‐Boichard

**Affiliations:** ^1^ Wageningen University & Research, Animal Breeding and Genomics Wageningen The Netherlands; ^2^ Facoltà di Scienze Agrarie, Alimentari e Ambientali/DIANA Dipartimento di Scienze Animali, Della Nutrizione e Degli Alimenti Università Cattolica del Sacro Cuore Piacenza Italy; ^3^ Facoltà di Scienze Agrarie, Alimentari e Ambientali, BioDNA Centro di Ricerca Sulla Biodiversità e Sul DNA Antico Università Cattolica del Sacro Cuore Piacenza Italy; ^4^ Dipartimento di Scienze Veterinarie Università Degli Studi di Messina Messina Italy; ^5^ GenPhySE, Université de Toulouse, INRAE, ENVT Castanet Tolosan France; ^6^ Universität für Bodenkultur Wien Austria; ^7^ Friedrich‐Loeffler‐Institut, Institute of Farm Animal Genetics Neustadt‐Mariensee Germany; ^8^ Department of Biomedical Sciences and Pathobiology, Animal Breeding and Genetics University of Veterinary Medicine Vienna Vienna Austria; ^9^ University of Minnesota College of Veterinary Medicine St Paul Minnesota USA; ^10^ Centre d'Anthropobiologie et de Génomique de Toulouse, CNRS UMR 5288 Université Paul Sabatier Toulouse France; ^11^ Trinity College Dublin University of Dublin Dublin Ireland; ^12^ Centre for Genetic Resources, the Netherlands (CGN) of Wageningen University & Research Wageningen The Netherlands; ^13^ Wageningen Bioveterinary Research Lelystad The Netherlands; ^14^ Université Paris‐Saclay, INRAE, AgroParisTech, GABI Jouy‐en‐Josas France; ^15^ Institute of Agricultural Biology and Biotechnology, National Research Council (IBBA‐CNR) Milan Italy

**Keywords:** biodiversity, farm animals, multispecies SNP array

## Abstract

Molecular genetic characterization of genetic resources is essential to study biodiversity. Whereas whole genome sequencing is still relatively expensive, low density SNP arrays offer a cost‐effective and standardized solution. However, most of the current arrays are species specific. Their high SNP density often exceeds diversity mapping requirements and remains too costly for many genetic resource managers. The IMAGE H2020 project aimed at developing a low‐cost multispecies SNP array to facilitate mapping of the genetic diversity in samples stored in gene banks and in vivo (on farm) traditional populations. This farm animal multispecies array contains approximately 10 K SNPs per species. The species included are cattle, sheep, goat, horse, pig, and chicken. We developed and tested this array on many samples from each of the six species. We describe here the SNP coverage and informativity across 253 breeds. We show that the array can be used to cluster local breeds according to history and genetic diversity. We illustrate its use for parentage testing. The array is publicly available at a reasonable price if ordered in multiples of 384 samples, leading to an overall cost of genotyping of approximately 15 euros per sample.

## INTRODUCTION

Animal gene banks host samples of a broad range of breeds of farm animals (Leroy et al. (2019)). These include traditional local breeds adapted to local environments, which may harbor variants important for disease resistance and adaptation‐related traits. Characterizing and comparing genome‐wide variation in animals from local populations is key to the identification of such variants and requires a cost‐efficient and universally usable single nucleotide polymorphism (SNP) genotyping system that includes the species of interest. Guidelines for molecular characterization of animal genetic resources have been recently updated (Ajmone‐Marsan et al., 2023). In farm animals, SNP arrays are available with medium to high marker density (BovineHD DNA Analysis Kit, 2022; BovineSNP50 DNA Analysis Kit, 2022; Groenen et al., 2012; Kijas et al., 2012; Kranis et al., 2013; McCue et al., 2012; Ramos et al., 2009; Schaefer et al., 2017; Tosser‐Klopp et al., 2014). There are two primary platforms—Illumina and Affymetrix—and numerous SNP panels are available for each species, with varying degrees of overlap between the marker sets depending on the species. The number of SNPs on these arrays for the major agricultural species such as cattle, sheep, goats, pigs, horses, and chickens, ranges from 25 k to 770 k. Furthermore, some additional arrays are not publicly accessible but owned by private companies or consortia. Most of these SNP panels have been designed for genotyping commercial populations to conduct QTL and genome‐wide association studies. These arrays are also relatively expensive to use and undergo changes over time, which makes cross‐study comparisons difficult. When inheritance or parentage verification or breed origin information is required, low density arrays provide sufficient information at a reduced cost whereas denser arrays often provide redundant data for these investigations.

Thus, low‐density SNP arrays, easily accessible worldwide without any restrictions, would be sufficient to support gene banking strategies. Moreover, the unqueried genotypic variation could be imputed to a higher SNP array, provided that a sufficient number of reference genotypes at a higher density are available for the breed. The low‐density SNP arrays can be used to monitor genetic variation in animal collections stored in vitro, such as in gene banks, as well as in in vivo populations. These datasets can be compared with publicly available datasets to make informed choices of which material should be stored in gene banks. When this information is shared globally across gene banks, a comprehensive overview can be obtained of the diversity conserved for each species, and in more detail for each breed. This facilitates monitoring of genetic variation across all species maintained in gene banks.

Gene bank collections harbor genetic variants that may be the target of selection in extant populations. When samples from gene banks are used for the reproductive management of a given population, it is crucial to know the molecular characteristics of the donor animal in relation to the amount of heterozygosity, variation in immune‐related genes, presence of disease‐susceptibility related alleles or known genetic defects. The H2020 project IMAGE was designed to address all such objectives through the development of a multispecies SNP array for the six main livestock species (cattle, sheep, goat, horse, pig, chicken) most represented in national gene banks. Scientific coordinators were identified in the IMAGE consortium for each species to centralize information on SNPs.

To have a complete picture of diversity, information on the autosomes, the sex chromosomes, and the mitochondrial DNA (mtDNA) is needed, due to the different inheritance patterns of these genetic markers (Várquez‐Miranda & Barker, 2021). In many cases, the SNP arrays are more biased towards the autosomes and commercial breeds. Therefore, an easily tractable molecular tool is needed. This tool should target variation across species in each of the three genetic components (autosomal, sex, and mitochondrial chromosomes), offer increased resolution in traditional breeds, and maintain sufficient overlap to current arrays for comparison with commercial breeds. To this end, the aim of this research was to design a 10‐K SNP panel including publicly available markers together with new loci specifically designed by IMAGE partners targeting functional variants, ancestral variants, and markers on sex chromosomes and mtDNA for each of the six species.

## MATERIALS AND METHODS

### Selection of candidate SNPs

For each species, candidate SNPs were collected from publicly available SNP panels. The SNP panels per species used are listed in Data S1. Information on the reference set for parentage testing, if publicly available per species, was included. Partners of IMAGE shared their genotyping data to identify highly informative markers with a minor allele frequency (MAF) >0.3 in multiple traditional populations/breeds, which provided 80% of the SNPs selected for the array. The minor allele frequency was mainly derived from genotyped in‐house collections. The remaining 20% were based on their relevance by species and their functionality i.e.: SNPs located on the sex chromosomes X/Y or Z/W (Colli et al., 2018; Felkel et al., 2019; Pariset et al., 2006; Wong et al., 2004); mtDNA variation covering the different haplogroups within each of the six species (Colli et al., 2018; Jansen et al., 2002; Pariset et al., 2011; Petersen et al., 2013; Upadhyay et al., 2017; Yang et al., 2017), ancestral SNPs (obtained either from ancient DNA sequencing, or comparing ancestral genomes of extant wild ancestral species); trait‐related markers (derived from OMIA; https://omia.org); major histocompatibility complex (MHC) variation (animal dbSNP; https://www.ncbi.nlm.nih.gov/snp/; Ali et al., 2017; Matukumalli et al., 2009), and supplemented by a random SNP in the coding region in a randomly selected gene located in QTL regions (animal QTL, www.animalgenome.org).

### Genotyping and quality criteria applied to candidate SNPs

The candidate SNPs (63 012 across species) were submitted to Affymetrix in 71‐mer format with the SNP provided on the forward strand at base pair position 36. Following in silico analysis, probes were classified as either ‘*recommended*’, ‘*neutral*’, ‘*not recommended*’, or ‘*not possible*’ based on their p‐convert value (probability of SNP conversion). P‐convert values were generated using Affymetrix power tool (APT) AxiomGTv1 algorithm to ensure a high‐quality final array (Roorkiwal et al., 2018). P‐convert values >0.6 signal a greater likelihood of successful probe assignment to each SNP on the array, considering factors such as probe sequence, binding energies, and effects from neighboring SNPs. Finally, a total of 59 726 SNPs qualified as either recommended or neutral, exhibiting a p‐convert score >0.6, were retained for genotyping evaluation. The genotyped SNPs were filtered following the manufacturer's ‘*best practices workflow*’ in Axiom Analysis Suite software (https://www.thermofisher.com/uk/en/home/technical‐resources/software‐downloads/axiom‐analysis‐suite.html). SNPs with dish quality control values >0.82, call rates >0.97 and an average call rate for passing samples >0.98 were retained in the final version of the array.

### Validation of high‐quality SNPs

Genomic DNA samples from animals representing a variety of backgrounds, either from gene banks or in situ populations of traditional breeds, were provided by IMAGE partners and their collaborators to be used for validation of the IMAGE001v1 array. DNA samples were either derived from blood or sperm samples. Species and breed origin of these animals are presented in Table S2. Within the porcine test panel, three trios were incorporated in the validation to validate the porcine SNP set for parentage testing. Genotyping of the animals from all six species was performed on the Affymetrix‐platform. In total, 1854 samples were gathered, spread across 241 breeds, and representing an average number of 309 samples per species. The allele frequency of the SNPs selected for traits (reference/alternative) was calculated per species as well as per breed, for all SNPs that produced a genotype. After a first step of validation, for each of the species, we replaced SNP markers that did not work, and finally produced a version 2 (IMAGE001v2), as described below. The newly selected SNPs per category i.e. MHC region, mtDNA, traits, sex chromosomes, and ancestral are indicated in detail in Table S2 (see IMAGE version column).

### Genetic analysis of genotypes

The estimation of accurate genetic relatedness among individuals provides valuable insights into lineage, heritable traits, and potential breeding strategies. We first performed principal component analyses using PLINKv1.07 (Purcell et al., 2007) on autosomal SNP genotypes to overview diversity across breeds within the swine species. In addition, we built a Neighbor‐Joining tree to investigate the genetic structure of chicken breeds where the data were filtered and formatted using plink 1.9. The subsequent dataset consisted of 6743 SNPs located on autosomes with a mean genotyping rate of 99.9%. An identity by distance pairwise matrix was computed between all individuals using the Hamming distance (function “—distance ‘1‐ibs’ «). This matrix was then used by the APE R package (Paradis & Schliep, 2019) to compute a neighbor‐joining tree (function « nj() »). This tree was plotted using the ggtree (Yu, 2020) R package.

Second, the extent and pattern of linkage disequilibrium (LD) was calculated. To determine the relatedness between individuals, we processed the dataset containing polymorphic SNPs to remove those in LD by employing a 50‐SNP sliding window approach and eliminating all variants with an *r* ^2^ of 0.3 or higher. We also excluded SNPs with an MAF <0.05 within breeds within a species and a genotype call rate below 90%. Using the refined dataset comprising 50 951 SNPs across the six species, we assessed relatedness among all animals across breeds within a species using PLINKv1.07 (Purcell et al., 2007).

To assess the reliability of our SNPs to infer parent–offspring relationships without prior relational information, we randomly selected 14 pigs and two pig trios including a boar, a sow, and their offspring. The approach implemented in the R package ‘apparent’ (Melo & Hale, 2019) was used to estimate the relationship between each pair of individuals. In brief, ‘apparent’ adopts a parentage analysis method based on genetic identity, through Gower distance, comparing the genetic identity of a hypothetical progeny (Pij) from a potential parent pair (i and j) at homozygous loci with all potential offspring (PHk) within a group of animals. When Gower distance is <0.05, it is considered evidence that the pair (i and j) is the true parent of offspring k. The significance of a trio (parental pair ij and offspring k) is ascertained by comparing its Gower distance to the Gower distance of the entire population (Melo & Hale, 2019).

## RESULTS

### SNP selection and validation of the SNPs included on the IMAGE001 array

The number of SNPs per species on the IMAGE001v1 array ranged from 8584 in chicken to 12 114 in sheep (Table 1). Detailed information on the categories of SNPs included in the IMAGE001v1 array of mappable markers is provided in Table S1. The SNP coverage across the genome for the six species is presented in Figure S1. The SNPs are spread uniformly across the chromosomes with some exceptions per species because of a higher coverage in QTL regions. The average distance between SNPs for the mammals was around 260 kb. Chickens had a higher SNP density with an average spacing of 120 kb between adjacent SNPs. In mammals, goats had the lowest density, with one SNP every 300 kb (Table S1).

**TABLE 1:** Overview of SNPs per species for the IMAGE001v1 array.

Species	Cattle	Sheep	Goat	Horse	Pig	Chicken
Reference genome	*Bos taurus*	*Ovis aries*	*Capra hircus*	*Equus caballus*	*Sus scrofa*	*Gallus gallus*
	UM3.1	Oar_v3.1	ARS1	EquCab3.0	Susscrofa 11.1	GRCg6a
Array information used	BovineHD	Ovine SNP50	CaprineHD SNP chip (.600 k)	670 k SNP array	Porcine60K	Chicken 60 k
	Bovine SNP50		GoatSNP50	EquineSNP50	Axiom_PigHDv.1 (>650 k)	ChickenHD 600 k
	Bovine LDv2.0					
SNPs						
Overlap existing arrays	7817^a^	9583^a^	7979^a^	7748^a^	6173^a^	6173^a^
Sex chromosome X/Z	240	134	12	80	26	100
Sex chromosome Y/W	5	2	69	0	36	96
mtDNA	13	136	170	0	36	90
Ancestral	974	256	1043	322	2000	1543
Trait	73	80	1164	50	167	20
MHC	134	0	0	203	9	63
Genes in QTL‐regions	1723	800	60	1537	1289	0
Total^b^	10 093	10 111	9993	10 114	10 107	9306

^a^ Number of SNPs derived from existing SNP arrays.

^b^ The total number of SNPs for each species might be lower than the sum of all SNPs in the column because an SNP from a public array might also be present among the ancestral SNPs, QTL regions, or any other category.

MHC, major histocompatibility complex; mtDNA, mitochondrial DNA.

### Validation of the SNPs included on the IMAGE001v1 array

Validation of the array was performed using in total 1854 DNA samples derived from 241 breeds of the six species (Table S2). The average number of breeds genotyped was 40.1 and ranged from 25 for pigs to 66 for sheep. The number of animals genotyped for the validation process varied from 159 in pigs to 527 in sheep. The number of animals selected per breed over the six species was on average 7.3 and varied from 1 to 37. Of the 59 726 SNPs included on the Affymetrix IMAGE001v1 array, 50 951 SNPs were successfully genotyped, representing 85.3% of the total (Table 2). The proportion of SNPs successfully genotyped per species ranged from 81.7% in chickens to 89.4% in sheep. After validation, the SNPs that did not work were replaced by new SNPs to produce IMAGE001v2 array (Table S1).

**TABLE 2:** Validation results for IMAGE001v1 array.

Species	# SNPs on the IMAGE001v1 per species	# of SNPs passed genotype validation	% successful genotyped IMAGE001v1	Average distance between SNPs IMAGE001v1 (bp)
Cattle	10 093	8692	86.1	297 236
Sheep	10 111	9043	89.4	296 707
Goat	9993	8383	83.9	300 782
Horse	10 114	8523	84.3	266 957
Pig	10 107	8699	86.0	290 073
Chicken	9308	7611	81.7	120 084
Total array	59 726	50 951	85.3	

### LD patterns between SNPs

**FIGURE 1:**
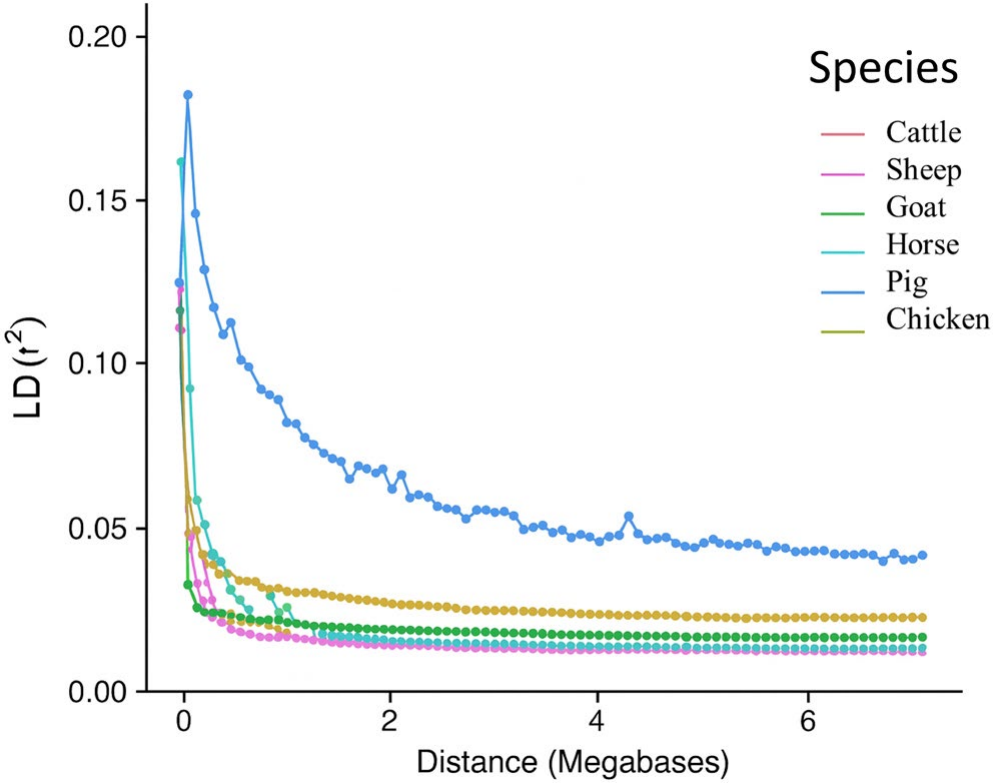
Plot of linkage disequilibrium (LD, *r* ^2^ [BovineSNP50 DNA Analysis Kit, 2022]) against distance between SNPs in the six species. Dots indicate observed pairwise LD. Lines show the decay of LD in the genome‐wide data. Each color represents a different species as indicated in the legend. Breeds with <5 animals were not included to improve readability.

The pairwise LD based on *r* (BovineSNP50 DNA Analysis Kit, 2022) values using the IMAGE001v1 data show similar patterns in the six species (Figure 1). Short‐range LD (*r* ^2^ < 0.2) is present between SNPs up to 0.5 Mb and drops with increasing distance. After 2 Mb, the *r* ^2^ in pigs remains around 0.05, displaying the highest extent of LD between SNPs, while in other species *r* ^2^ drops below 0.03.

### Within species populations cluster according to origin

**FIGURE 2:**
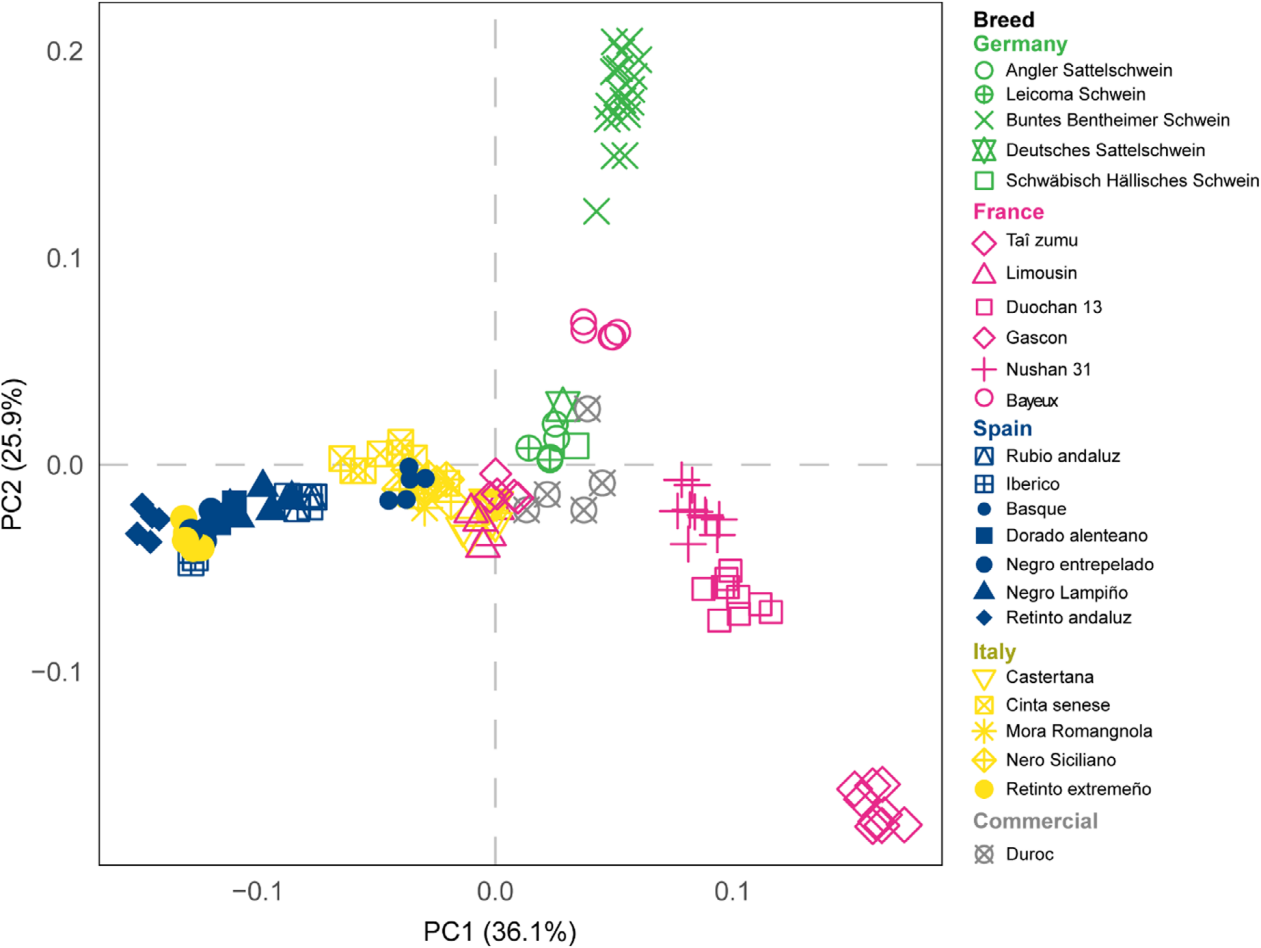
Principal component (PC) plot from 132 pigs from 13 breeds derived from 4 countries (Germany, France, Italy, and Spain). The breeds are ranked in alphabetical order with different symbols and colors. Breeds with <5 animals per breed and the experimental lines were removed to improve readability. Percentage values in the axis labels represent the percentage of the total variance explained by the given PC.

With the selected candidate SNPs, we were able to capture the population differentiation in the six species. The principal component (PC) analysis result for pig (Figure 2) shows a subset of breeds chosen from four countries (Germany, France, Italy, and Spain). The first two principal components explain 62% of total variance. The commercial breed Duroc (from Spain) is located in the center of the plot. The breeds tend to separate according to country of origin and the distribution of the animals fits well with the known history of the breeds (Poklukar et al., 2023). For example, local breeds from Spain (Negro Lampiño, Negro Entrepelado and Basque), France (Bayeux), and Germany (Buntes Bentheimer) are clearly isolated. The Tai zumu is a Sino‐European synthetic line developed in France that is clearly separated from all others in PC1, as expected from its known background. The PC analysis plots for the remaining five species exhibit similar patterns (Figure S2A–E).

**FIGURE 3:**
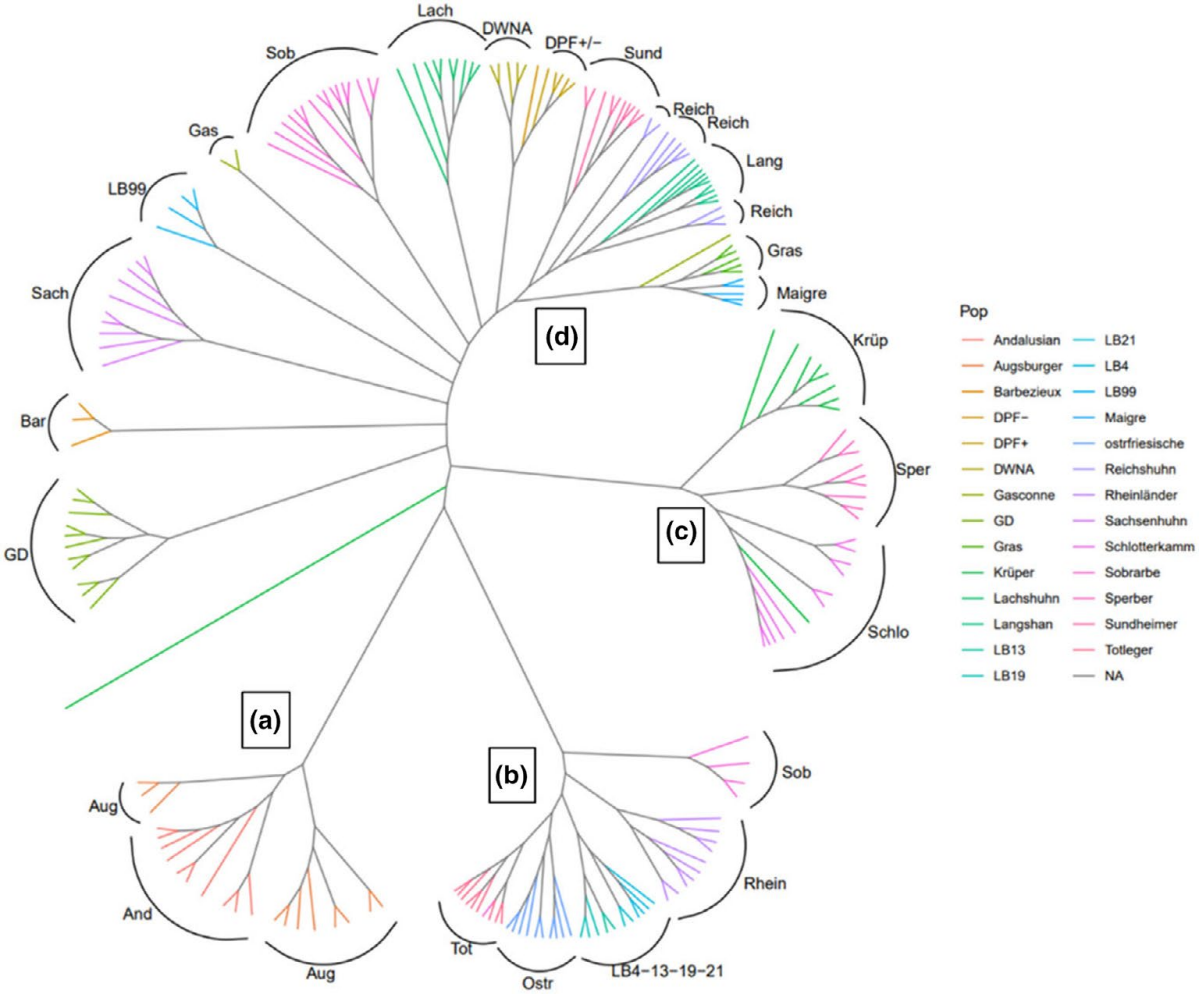
Neighbor‐Joining tree of the chicken populations (branches A, B, C, D are discussed in the text).

In chickens, genotypes were obtained for 204 samples from 18 local breeds and nine experimental lines provided by Germany, France, and Spain. The MAF across population is 0.34, showing that the array is informative over a large range of populations. Breeds tend to cluster according to their history rather than their country, which shows the interest of sharing information across gene banks to assess their complementarity. Another way of representing diversity between the chicken populations is by using a Neighbor‐Joining tree as presented in Figure 3. The chicken Neighbor‐Joining tree features 4 main branches: branch A includes only one Spanish breed (Blue Andalusian) and one German breed (Augsburger); branch B includes three German breeds (Rheinlander, Westfaelische Totleger, and Ostfriesische Moewen), and the four congenic lines which are all of White Leghorn origin; branch C includes three German breeds (Krüper, Deutsche Sperber, and Bergische Schlotterkamm); branch D includes the remaining German breeds, most animals of the Gallina del Sobrarbe, the French local breeds and the experimental lines of a non‐White Leghorn origin. The divergent lines such as Fat/Lean lines and DPF+/DPF− cluster together.

### The IMAGE001 SNP array as tool to estimate relationship between individuals

**FIGURE 4:**
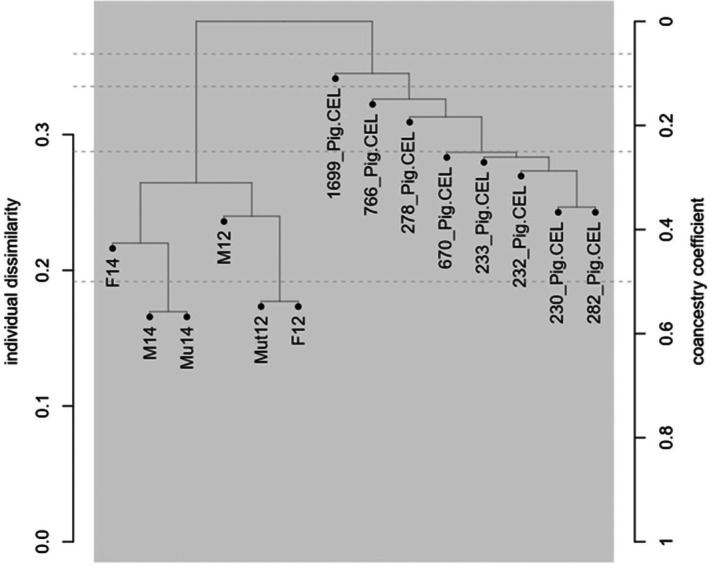
Assessment of parent–offspring relationships among selected porcine samples using the IMAGE001v1 SNP Array. This figure illustrates the successful identification and separation of two porcine trios from a group of unrelated pigs, demonstrating the efficacy of the array in determining familial associations. M denotes male, F denotes female, Mu and Mut indicate offspring. The numbers 12 and 14 represent the respective families to which they belong.

To test the reliability of the array to infer parent–offspring relationships without prior pedigree information, we randomly selected 14 pigs and two pig trios including a boar, a sow, and one offspring. The two trios were separated from the 14 unrelated pigs and the individuals of the trios were correctly assigned to the correct family (Figure 4). These results indicate that the SNPs present on the array can be used to extract family relationships between individuals and populations. We evaluated the degree of relationship between animals with the Gower distance (Melo & Hale, 2019). This approach delivered direct parent–offspring associations by assigning the only two significant trios belonging to our prior families (Table 3).

**TABLE 3:** Direct parent–offspring associations identified using the Gower distance (GD) in the 'apparent' application.

Parent1^a^	Parent2^a^	Offspring^a^	Cross.Type	SNPs	GD	*p* value
F14	M14	Mu14	Outcross	1445	0.014	<0.01
F12	M12	Mut12	Outcross	1470	0.022	<0.01

^a^ F denotes female, M denotes male, Mu and Mut are offspring. The numbers 12 and 14 represent the respective families to which they belong.

### SNPs associated with economically relevant traits

On the array, we included SNPs associated with known relevant traits in livestock for informed decision‐making about gene bank specimen reuse based. Of the 13 398 initially selected SNPs related to traits, a total of 12 385 could be assayed using the multispecies array IMAGE001v1. However, probe design was not feasible or recommended for 1013 trait related markers, due to a low design score because of either a high number of SNPs in the flanking region (especially in the MHC) or the strongly biased base composition of the flanking sequences. Additionally, several SNPs were selected directly from sources such as OMIA or literature, which may result in an underrepresentation of the evaluated breed among our genotyped individuals. Of the functional trait SNPs, 101 have been classified as causal mutations. Further information on the markers for each species, including trait information and allele frequency, can be found in Table S1. The causal SNP information can be used, for instance, to increase or decrease the frequency of a specific SNP, or to plan specific matings to favor or not the production of homozygous carriers, and thus enhance or reduce the occurrence of the associated trait.

The animals genotyped for trait markers were not specifically chosen to encompass the whole range of phenotypic traits. This implies that a significant portion of the genotypes for the underlying trait markers may exhibit no variation. For chicken, we initially proposed 12 candidate copy number variations (CNVs). However, only three of these CNVs were successfully detected, and their inclusion collectively required more than 900 SNPs on the array. Despite CNVs potentially playing an important role in genomic studies, their genotyping through SNP arrays, particularly for species such as chickens, is fraught with challenges (Wolc et al., 2018). The limited capacity of these arrays is rapidly taken up by the multiple probes required for precise CNV detection, thereby leaving diminished space for other possibly informative SNPs. Consequently, we opted to exclude the CNV and instead incorporated 298 SNP‐associated traits into the array (Table S1). Examples of two trait‐related markers for horses are shown in Table 4 and pertain to the cream dilution and pearl coat colors within the *SLC45A2* gene.

**TABLE 4:** Missense SNPs related with coat color in horses.

SNP information		Genotype (*SLC45A2*)			SNP functional impact		Reference
AFFy ID	SNP pos	GG	GA	AA	Trait	Breed(s)	OMIA ID
Affx‐102 471 109	21:31690653	517	3	2	Coat color, cream dilution	–	OMIA 001344–9796
Affx‐1 002 475 697	21:31709690	522	1	0	Coat color, pearl	American Paint Horse Lusitano Purebred Spanish horse Quarter Horse	OMIA 001344–9796

### Mitochondrial DNA SNP validation

**FIGURE 5:**
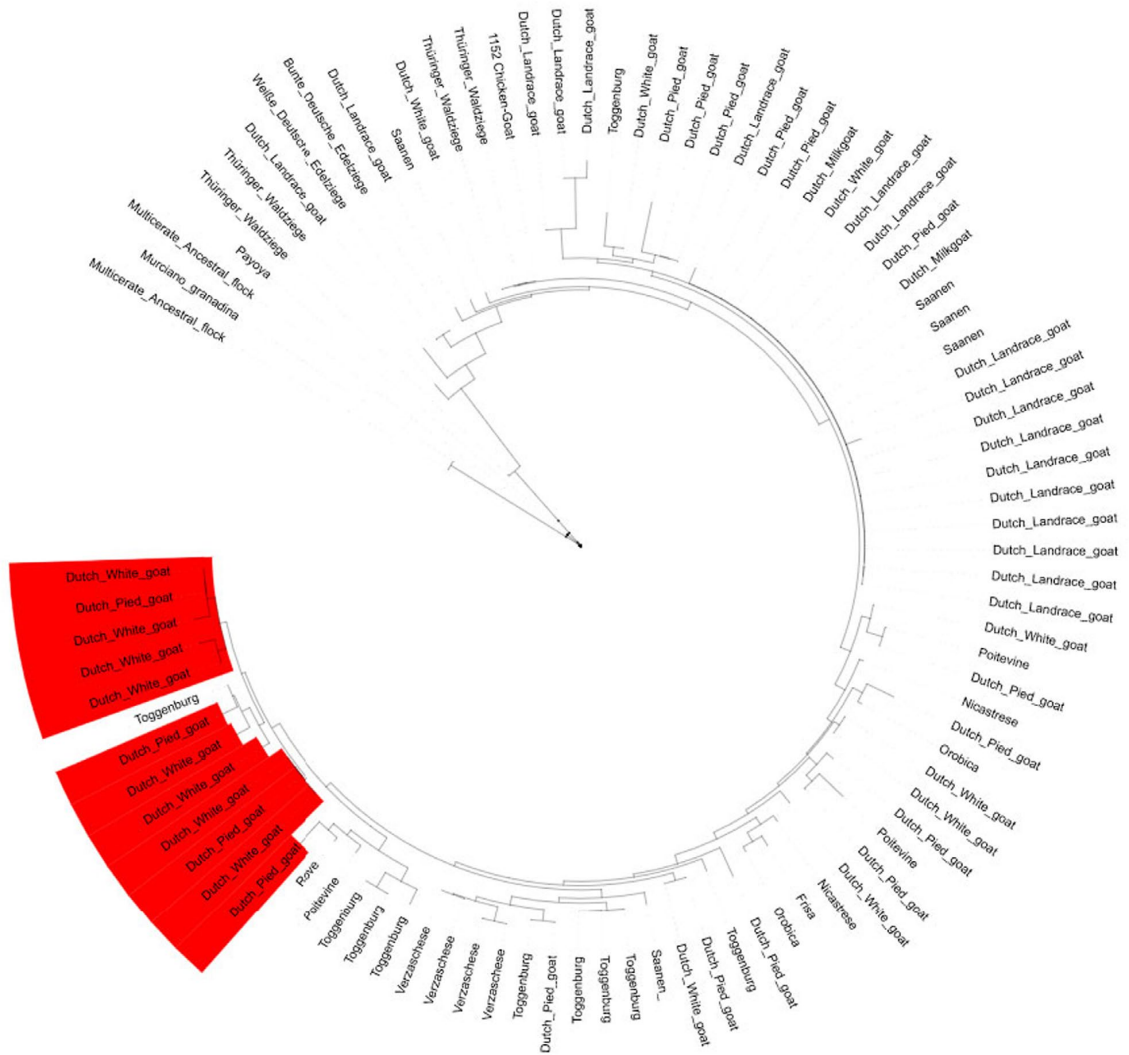
The caprine maximum‐likelihood phylogenetic tree using 207 animals representing 32 breeds and 147 markers of the mitochondrial DNA.

The mtDNA SNPs from goat were used as an example to build a caprine mtDNA phylogenetic tree (Figure 5). Of the 170 markers included on the array, 147 SNPs worked and gave a genotype for 207 goats from 32 breeds. The number of informative markers was still low, which led to a poor breed resolution in the maximum likelihood phylogenetic tree. Variation in the mtDNA explains only a very small part of the biodiversity and only documents the maternal lineages. Because this type of variation was frequently used for several species in the past, we included these variants to link to earlier studies (Harrison, 1989). Despite the limitations of mtDNA markers, these SNPs on the IMAGE001v1 array highlight its ability to compare maternal lineages with results provided by other studies. Comparison of results between markers differing by their inheritance pattern (either autosomal, sex‐linked, or maternally derived) makes it a valuable tool for understanding genetic relationships among breeds. For instance, maternal introgression may be revealed in the case of crosses between local breeds and exotic lines. It must be noted that in chickens, mtDNA and W chromosome follow the same transmission pattern and will both reveal any maternally derived introgression.

### Improvement of IMAGE001v1 to IMAGE001v2

In total, 8775 markers across the six species that did not perform on the IMAGE001v1 array were replaced with new markers from several categories leading to the IMAGE001v2 array. The total number of SNPs on the IMAGE001v2 array is 58 844. The final overview of the markers on the IMAGE001v2 array per species is given in Table 5. The SNPs listed in Table S1 selected for the IMAGE001v1 are indicated within column IMAGE V1 whereas the final list of SNPs on IMAGE001v2 including replaced SNPs are indicated in column V2. All SNPs on the IMAGE001v1 and v2 arrays presented in the table, including genome location and SNP type category. Further genotyping performed with final IMAGE001v2 array on sheep, horse, pig, and chicken, yielded a proportion of 2–5% of failed markers (personal communication: Michelle Tixier‐Boichard; INRAe, France). The rate varied according to the species and the batch of samples, chicken showing the highest value (Tixier‐Boichard, personal communication). The IMAGE001v2 array was not further validated in the present study and is commercially available (Thermo Fisher Scientific).

**TABLE 5:** Overview of SNPs per species for the final IMAGE001v2 array.

Species	Cattle	Sheep	Goat	Horse	Pig	Chicken
Reference genome	*Bos taurus*	*Ovis aries*	*Capra hircus*	*Equus caballus*	*Sus scrofa*	*Gallus gallus*
	UM3.1	Oar_v3.1	ARS1	EquCab3.0	Susscrofa 11.1	GRCg6a
Array information used	BovineHD	Ovine SNP50	CaprineHD SNP chip (.600 K)	670‐K SNP array	Porcine60K	Chicken 60 K
	Bovine SNP50		GoatSNP50	EquineSNP50	Axiom_PigHDv.1 (>650 K)	ChickenHD 600 K
	Bovine LDv2.0					
SNPs						
Overlap existing arrays	8564^a^	9583^a^	8904^a^	7071^a^	7967^a^	5384^a^
Sex chromosome X/Z	175	89	0	266	372	991
Sex chromosome Y/W	19	96	57	200	15	64
mtDNA	6	98	147	0	13	86
Ancestral	944	700	398	253	>90%	925
Trait	299	80	1815	32	152	298
MHC	63	0	0	47	0	0
Genes in QTL‐regions	0	2889	135	1374	1040	0
Total^b^	9808	12 114	10 616	8931	8791	8584

^a^ Number of SNPs derived from existing SNP arrays.

^b^ The total number of SNPs for each species might be lower than the sum of all SNPs in the column because a SNP from a public array might also be present among the ancestral SNPs, QTL regions, or any other category.

## DISCUSSION

### Applications in gene bank management and biodiversity conservation

Here, we describe the development and improvement of a multispecies SNP array for the main six livestock species bred worldwide: cattle, sheep, goat, horse, pig, and chicken. The final IMAGE001v2 array is an Affymetrix array, especially designed to genotype and compare animal material of traditional/local breeds stored in gene banks or in situ collections across the world. The inclusion of ancestral SNPs decreases the ascertainment bias and increases the probability that the array will be informative in any domestic population, regardless of its geographic origin. The excellent call rate of the selected SNPs highlights the reliability of the techniques used for capturing genetic information. We also show that the array can be used to exploit information from markers following different inheritance patterns (autosomal, sex‐linked, mtDNA).

The diversity plots supported the effectiveness of the chosen SNPs in distinguishing breeds and identifying within‐species population structure. These findings hold great potential for future research, including genetic characterization and the development of targeted breeding and conservation programs. The low‐density SNP genotypes can also be imputed to higher SNP densities if a sufficient number of reference samples genotyped at higher density is available. For all species there is an HD chip available except for goats where there is only a 50‐K array.

The developed array includes SNPs related to known phenotypes, immune traits and genetic defects, which add functional diversity to neutral diversity for characterizing populations and investigating heterozygosity and relatedness between animals. SNPs associated with diseases susceptibility and other health related traits allow selection of candidate animals for breeding programs whilst maintaining biodiversity in small breeds. It is expected that more traits will be unraveled in the near future, which will give more causal markers that can be explored that can be added to these arrays. By offering valuable genotype information, the array can guide decision‐making in gene bank specimen reuse, conservation strategies, and breeding programs. The knowledge of trait‐associated SNPs can help breeders select animals with desirable traits, leading to more efficient programs and improved livestock populations. Once current in situ populations have been genotyped, effective decisions can be made for sampling animals for cryo‐conservation.

### Perspectives

Potential future updates of the IMAGE001v2 array per species could be organized through the Molecular Markers and Parentage Testing Committee of the International Society of Animal Genetics (ISAG). Our results suggest a promising application of the IMAGE001 SNP array in determining relationships between animals. However, the sample size of the testing panel might not fully represent the array's performance in larger, more diverse populations. Future research could expand on this study by incorporating additional samples from more diverse origins to better understand its overall effectiveness and applicability. Coordination of users of the arrays can help to reduce the price of the arrays, since discounts are offered by Thermo Fisher Scientific for buying more than five arrays at a time.

In conclusion, the IMAGE array constitutes a helpful resource for the management of diversity within species as well as within breeds and for more advanced analyses and applications, including genome‐wide association studies (Sharmaa et al., 2015). It will facilitate assessment of genetic originality of traditional breeds to either supplement gene banks or to avoid the uploading of redundant samples. If all gene bank entries of the six species worldwide were genotyped with IMAGE001 array, a comprehensive molecular genetics overview would be obtained.

## AUTHOR CONTRIBUTIONS

R.P.M.A.C., R.G.P., and M.T.B. were responsible for the experimental design of the study. R.P.M.A.C. and S.J.H. were responsible for the generation and acquisition of genotyped material. The species teams responsible for marker selection are for the: (1) Bovine team: R.P.M.A.C., R.G.P., and D.B.; (2) Ovine team: M.D.C., E.S., M.B., L.C., and P.A.M.; (3) Caprine team: G.T.K., G.M., A.S., and L.C.; (4) Horse team: R.G.P., M.E.Mc.C., B.W., and L.O.; (5) Porcine team: D.J.S., R.G.P., R.P.M.A.C., and M.A.M.G.; (6) Chicken team: R.P.M.A.C., R.G.P., M.T.B., S.W., and N.P. R.G.P. and R.P.M.A.C. performed the bioinformatic analysis. R.P.M.A.C. and R.G.P. wrote the paper. All authors read, contributed to, and approved the manuscript.

## FUNDING INFORMATION

The research presented in this publication was funded within the IMAGE project, which received funding from the European Union's Horizon 2020 Research and Innovation Programme under the grant agreement n° 677353. Ludovic Orlando has received funding from the European Research Council (ERC) under the European Union's Horizon 2020 Research and Innovation Programme (grant agreements 681605‐PEGASUS, and 101071707‐HorsePower).

## CONFLICT OF INTEREST STATEMENT

None of the authors of this paper has a financial or personal relationship with other people or organizations that could inappropriately influence or bias the content of this paper.

## Supporting information


Data S1.

Table S1.

## Data Availability

Genotype data will be available on request.
