# Supplementary material for: IMAGE001: A new livestock multispecies SNP array to characterize genomic variation in European livestock gene bank collections
Source: Anim Genet. 2025 Sep 18;56(5):e70039. doi: 10.1111/age.70039 (PMC12445162; doi:10.1111/age.70039)
Supplement: Supplementary file 1 — Data S1. [file AGE-56-0-s002.docx]

**IMAGE001: a new livestock multispecies SNP array to characterize genomic variation in European livestock gene bank collections**


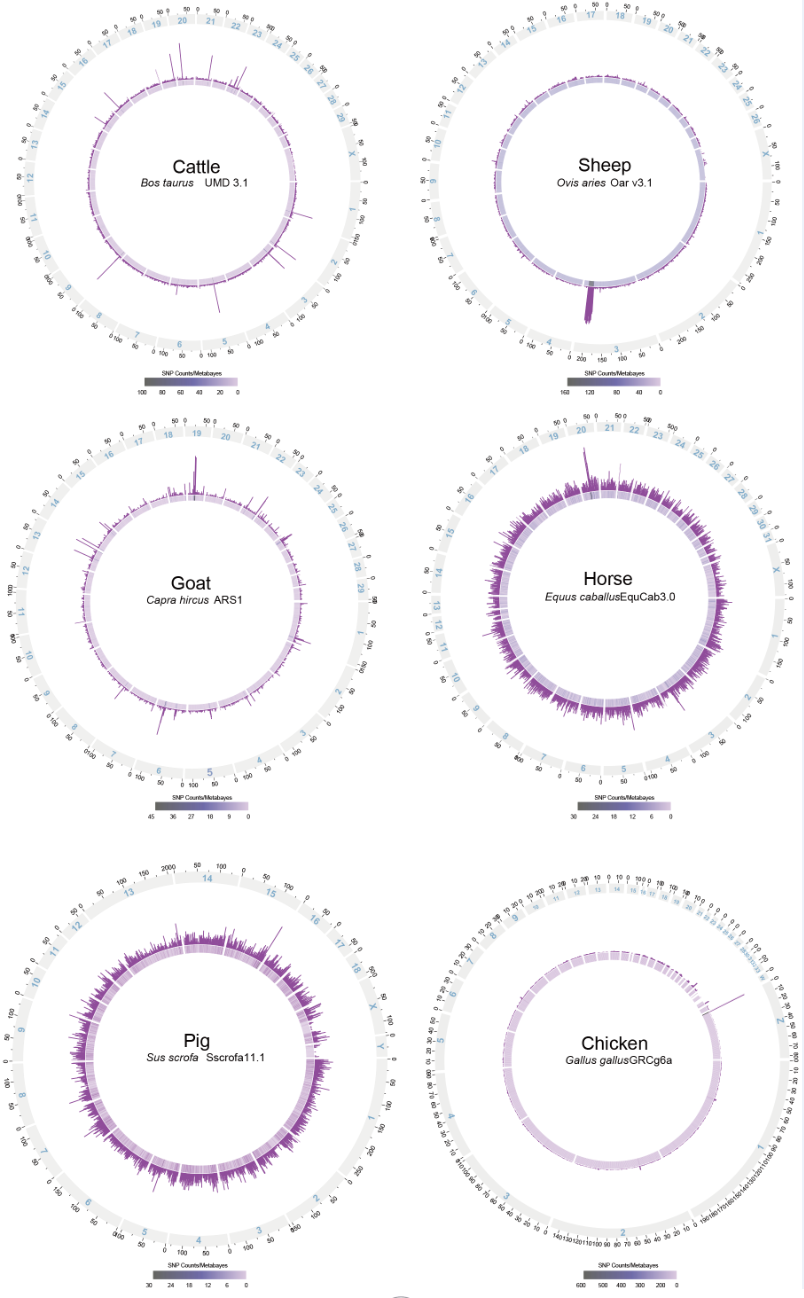


**Figure S1**. The coverage on the SNPs from IMAGE001v1 on the genome of the six species: A) cattle, B) sheep C) goat, D) horse E) pig and F) chicken.


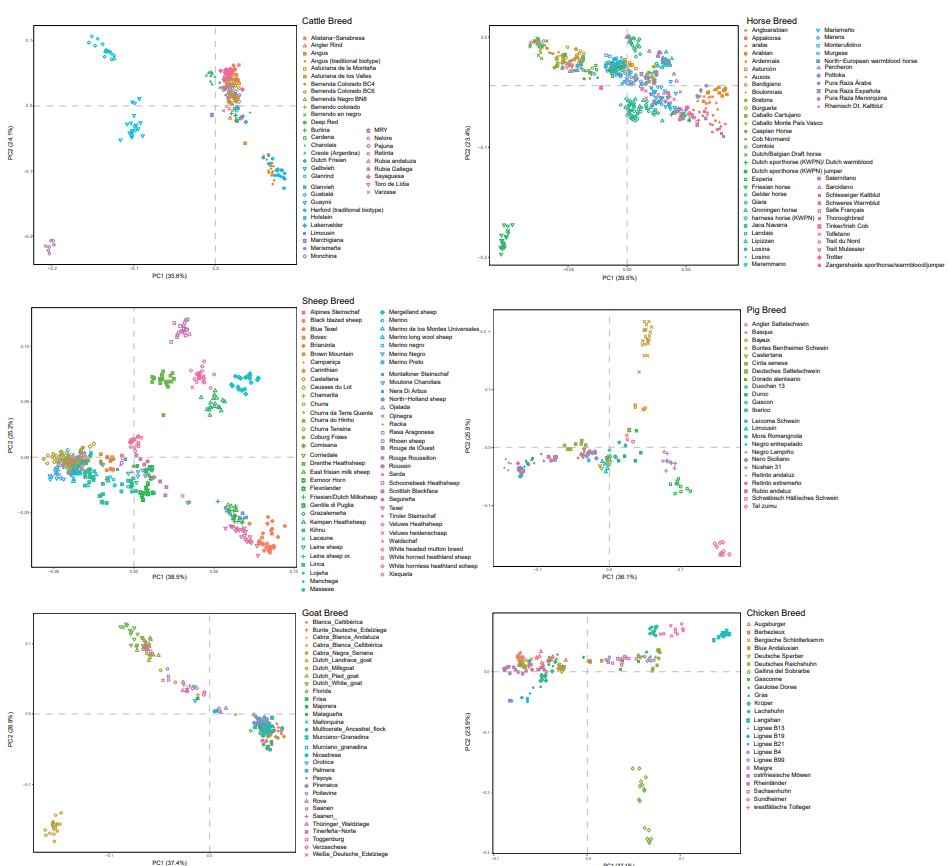


**Figure S2.** The PCA plot based on the SNPs from IMAGE001.V1 of the six species: cattle, horse, sheep, pig, goat and chicken. Breeds are given in alphabetic order and ranked on alphabetic order with different symbols and colours. Percentage values in the axis labels represent the percentage of the total variance explained by the given PC.

# Table S2. Detailed information regarding the number of animals per breed and per species evaluated in the IMAGE1 array

| **Species** | **Breed name** | **Samples** | **breed** |
| --- | --- | --- | --- |
|  |  |  | **category** |
|  | Berrenda Colorado BC4 | 1 | experimental |
|  | Berrenda Colorado BC6 | 1 | experimental |
|  | Berrenda Negro BN8 | 1 | experimental |
|  | Groninger White Headed | 1 | local |
|  | Deep Red | 1 | local |
|  | Charolais | 1 | transboundery |
|  | Dutch Friesian | 1 | local |
|  | Holstein-Friesian | 1 | transboundery |
|  | Dutch Belted | 1 | local |
|  | Marchigiana | 1 | local |
|  | Marismena | 1 | local |
|  | Meuse-Rhine -Yssel (MRY) | 2 | local |
|  | Rubia Gallega | 1 | local |
|  | Gelbvieh | 2 | local |
|  | Angler | 3 | transboundery |
|  | Glanrind | 4 | local |
|  | Limousin | 3 | transboundery |
|  | Burlina | 4 | local |
|  | Berrendo Colorado | 5 | local |
|  | Berrendo en Negro | 5 | local |
|  | Cardena | 5 | local |
|  | Pajuna | 5 | local |
|  | Retinta | 5 | local |
|  | Rubia Andaluza | 5 | local |
|  | Toro de Lidia | 5 | local |
|  | Varzese | 5 | local |
|  | Nelore | 9 | transboundery |
|  | DSN | 11 | local |
|  | Angus | 23 | transboundery |
|  | Asturiana de la Montana | 15 | local |
|  | Asturiana de los Valles | 15 | local |
|  | Guabala | 16 | local |
|  | Alistana-Sanabresa | 20 | local |
|  | Guaymi | 20 | local |
|  | Monchina | 20 | local |
|  | Sayaguesa | 20 | local |
|  | Creole (Argentina) | 21 | local |
|  | Hereford | 21 | transboundery |
| **Cow (total)** | **38 Breeds** | **281** |  |
| **Sheep** | Alpines_Steinschaf | 2 | local |
|  | Brianzola | 2 | local |
|  | Carinthian | 2 | local |
|  | Churra do Hinho | 2 | local |
|  | Coburg Foxes | 2 | local |
|  | Merino long wool sheep | 2 | local |
|  | Merino_Preto | 2 | local |
|  | Montafoner Steinschaf | 2 | local |
|  | Racka | 2 | local |
|  | Rhoen sheep | 2 | local |
|  | Rouge Roussillon | 2 | local |
|  | Waldschaf | 2 | local |
|  | White Headed Mutton | 2 | local |
|  | Bovec | 3 | local |
|  | Brown Mountain | 3 | local |
|  | Campaniça | 3 | local |
|  | Causses du Lot | 3 | local |
|  | Corriedale | 3 | local |
|  | Gentile di Puglia | 3 | local |
|  | Lacaune | 3 | local |
|  | Moutons Charollais | 3 | local |
|  | Rouge de louest | 3 | local |
|  | Roussin | 3 | local |
|  | Tiroler Steinschaf | 3 | local |
|  | Churra da Terra Quente | 4 | local |
|  | Comisana | 4 | local |
|  | Exmoor Horn | 4 | local |
|  | Kihnu | 4 | local |
|  | Massese | 4 | local |
|  | Nera Di Arbus | 4 | local |
|  | Sarda | 4 | local |
|  | Scottish Blackface | 4 | local |
|  | Grazalemena | 5 | local |
|  | Leine sheep | 5 | local |
|  | Lojena | 5 | local |
|  | Merino Negro | 5 | local |
|  | North-Holland sheep | 5 | local |
|  | East Frisian milk sheep | 7 | local |
|  | Veluwe Heath | 7 | local |
|  | Blue Texel | 8 | local |
|  | White horned heathland sheep | 8 | local |
|  | White hornless heathland scheep | 9 | local |
|  | Chamarita | 10 | local |
|  | Churra Tensina | 10 | local |
|  | Flevolander | 10 | local |
|  | Manchega | 10 | local |
|  | Merino de los Montes Universales | 10 | local |
|  | Merino Negro | 10 | local |
|  | Ojalada | 10 | local |
|  | Ojinegra | 10 | local |
|  | Rasa_Aragonesa | 10 | local |
|  | Segurena | 10 | local |
|  | Xisqueta | 10 | local |
|  | Bentheimer Landschaf | 11 | local |
|  | Merino | 11 | transboundery |
|  | Friesian Milk | 19 | local |
|  | Black Blazed | 20 | local |
|  | Drenthe_Heath | 20 | local |
|  | Kempen Heathsheep | 20 | local |
|  | Mergelland | 20 | local |
|  | Schoonebeek | 20 | local |
|  | Veluwe Heathsheep | 20 | local |
|  | Castellana | 21 | local |
|  | Texel | 21 | transboundery |
|  | Churra | 22 | local |
|  | Linca | 37 | local |
| **Sheep total** | **66 breeds** | **527** |  |
| **Goat** | Bunte Deutsche Edelziege | 1 | local |
|  | Frisa | 1 | local |
|  | Mallorquina | 1 | local |
|  | Palmera | 1 | local |
|  | Weiβe Deutsche Edelziege | 1 | local |
|  | Dutch Dairy | 2 | composite |
|  | Nicastrese | 2 | local |
|  | Orobica | 2 | local |
|  | Rove | 2 | local |
|  | Poitevine | 3 | local |
|  | Tinerfena-Norte | 3 | local |
|  | Majorera | 4 | local |
|  | ThUringer_Waldziege | 4 | local |
|  | Cabra Blanca Andaluza | 5 | local |
|  | Cabra Blanca Celtib├⌐rica | 5 | local |
|  | Cabra Negra Serrana | 5 | local |
|  | Payoya | 5 | local |
|  | Saanen | 8 | transboundery |
|  | Verzaschese | 5 | local |
|  | Malaguena | 6 | local |
|  | Murciano-Granadina | 14 | local |
|  | Florida | 9 | local |
|  | Toggenburg | 11 | local |
|  | Blanca Celtib├⌐rica | 13 | local |
|  | Dutch Pied | 17 | local |
|  | Dutch White | 18 | local |
|  | Dutch Landrace | 19 | local |
|  | Multicerate Ancestral | 20 | local |
|  | Pirenaica | 20 | local |
| **Goat total** | **29 Breeds** | **207** |  |
| **Horses** | Schweres Warmblut | 2 | local |
|  | Schleswiger Kaltblut | 1 | local |
|  | Rheinisch Dt. Kaltblut | 1 | local |
|  | Marismeño | 10 | local |
|  | Burguete | 6 | local |
|  | Caballo Deporte Español | 5 | local |
|  | Caballo Monte País Vasco | 6 | local |
|  | Hispano Árabe | 5 | local |
|  | Jaca Navarra | 5 | local |
|  | Pura Raza Menorquina | 5 | local |
|  | Pottoka | 5 | local |
|  | Pura Raza Árabe | 5 | local |
|  | Pura Raza Española | 5 | local |
|  | Caballo Cartujano | 5 | local |
|  | Losino | 6 | local |
|  | Losina | 20 | local |
|  | Angloarabian | 15 | local |
|  | Bardigiano | 15 | local |
|  | Esperia | 15 | local |
|  | Giara | 15 | local |
|  | Lipizzan | 15 | transboundery |
|  | Maremmano | 15 | local |
|  | Monterufolino | 15 | local |
|  | Murgese | 15 | local |
|  | Arabian | 10 | transboundery |
|  | Salernitano | 5 | local |
|  | Thoroughbred | 4 | transboundery |
|  | Sarcidano | 15 | local |
|  | Tolfetano | 15 | local |
|  | Asturcón | 15 | local |
|  | Gelder horse | 18 | local |
|  | Groningen horse | 20 | local |
|  | Dutch sporthorse (KWPN)/ Dutch warmblood | 5 | transboundery |
|  | Belgian Draft | 20 | transboundery |
|  | harness horse (KWPN) | 16 | transboundery |
|  | Friesian horse | 20 | local |
|  | C_H | 1 | composite |
|  | North-European warmblood horse | 1 | transboundery |
|  | Dutch sporthorse (KWPN) - jumper | 3 | transboundery |
|  | Trotter | 2 | local |
|  | Caspian horse | 2 | local |
|  | Tinker/Irish Cob | 1 | local |
|  | Appaloosa | 1 | local |
|  | Zangersheide sporthorse/warmblood/jumper | 1 | local |
|  | Ardennais | 4 | local |
|  | Auxois | 4 | local |
|  | Boulonnais | 8 | local |
|  | Breton | 9 | local |
|  | Cob Normand | 11 | local |
|  | Comtois | 3 | local |
|  | Landais | 2 | local |
|  | Merens | 2 | local |
|  | Percheron | 5 | local |
|  | Trait Mulassier | 4 | local |
|  | Selle Français | 9 | local |
|  | Trait du Nord | 3 | local |
|  | Arabe | 18 | local |
| **Horse total** | **57 Breeds** | **469** |  |
| **Pig** | Schwäbisch Hällisches Schwein | 2 | local |
|  | Angler Sattelschwein | 4 | local |
|  | Buntes Bentheimer | 20 | local |
|  | Leicoma Schwein | 2 | local |
|  | Deutsches Sattelschwein | 1 | local |
|  | Cerdo Iberico | 7 | local |
|  | Duroc | 5 | transboundery |
|  | Rubio andaluz | 5 | local |
|  | Dorado alenteano | 5 | local |
|  | Negro entrepelado | 6 | local |
|  | Negro Lampiño | 6 | local |
|  | Retinto andaluz | 5 | local |
|  | Retinto extremeño | 6 | local |
|  | Basque | 5 | local |
|  | Bayeux | 5 | local |
|  | Duochan 13 | 10 | local |
|  | Gascon | 5 | local |
|  | Limousin | 5 | local |
|  | Nushan 31 | 10 | local |
|  | PBO | 5 | composite |
|  | Taî zumu | 9 | composite |
|  | Castertana | 10 | local |
|  | Cinta senese | 10 | local |
|  | Mora Romangnola | 1 | local |
|  | Nero Siciliano | 10 | local |
| **Pig total** | **25 breeds** | **159** |  |
| **Chicken** | Lignee B13 (MHC) | 2 | experimental |
|  | Lignee B21 (MHC) | 2 | experimental |
|  | Barbezieux | 3 | local |
|  | Gasconne | 3 | local |
|  | Lignee B19 (MHC) | 3 | experimental |
|  | DPF-(low duration of fertility line) | 4 | composite |
|  | Lignee B4 (MHC) | 4 | experimental |
|  | DPF+ (high duration of fertility line) | 5 | experimental |
|  | DWNA (dwarf naked neck layer) | 5 | composite |
|  | Gras (high abdominal fatness) | 5 | composite |
|  | Lignee B99 (Bresse breed) | 5 | experimental |
|  | Maigre (Low abdominal fatness) | 5 | composite |
|  | Blue Andalusian | 10 | local |
|  | Deutsches Reichshuhn | 20 | local |
|  | Krüper | 10 | local |
|  | Langshan | 10 | local |
|  | Ostrfriesische Möwe | 10 | local |
|  | Rheinländer | 10 | local |
|  | Sachsenhuhn | 10 | local |
|  | Sundheimer | 10 | local |
|  | Westfälische Totleger | 10 | local |
|  | Augsburger | 11 | local |
|  | Bergischer Schlotterkamm | 11 | local |
|  | Deutsche Sperber | 11 | local |
|  | Gauloise Doree | 12 | local |
|  | Gallina del Sobrarbe | 20 | local |
| **Chicken total** | **26 breeds** | **211** |  |
